# Polygenic resilience score may be sensitive to preclinical Alzheimer’s disease changes

**Published:** 2023

**Authors:** Jaclyn M. Eissman, Greyson Wells, Omair A. Khan, Dandan Liu, Vladislav A. Petyuk, Katherine A. Gifford, Logan Dumitrescu, Angela L. Jefferson, Timothy J. Hohman

**Affiliations:** 1 Vanderbilt Memory and Alzheimer’s Center, Vanderbilt University Medical Center, Nashville, TN 37212, USA; 2 Vanderbilt Genetics Institute, Vanderbilt University Medical Center, Nashville, TN 37212, USA; 3 Department of Biostatistics, Vanderbilt University Medical Center, Nashville, TN 37212, USA; 4 Biological Sciences Division and Environmental Molecular Sciences Laboratory, Pacific Northwest, National Laboratory, Richland, WA 99354, USA

**Keywords:** Alzheimer’s disease, polygenic risk, resilience, preclinical, cognition

## Abstract

Late-onset Alzheimer’s disease (LOAD) is a polygenic disorder with a long prodromal phase, making early diagnosis challenging. Twin studies estimate LOAD as 60–80% heritable, and while common genetic variants can account for 30% of this heritability, nearly 70% remains “missing”. Polygenic risk scores (PRS) leverage combined effects of many loci to predict LOAD risk, but often lack sensitivity to preclinical disease changes, limiting clinical utility. Our group has built and published on a resilience phenotype to model better-than-expected cognition give amyloid pathology burden and hypothesized it may assist in preclinical polygenic risk prediction. Thus, we built a LOAD PRS and a resilience PRS and evaluated both in predicting cognition in a dementia-free cohort (N=254). The LOAD PRS had a significant main effect on baseline memory (β=−0.18, P=1.68E-03). Both the LOAD PRS (β=−0.03, P=1.19E-03) and the resilience PRS (β=0.02, P=0.03) had significant main effects on annual memory decline. The resilience PRS interacted with CSF Aβ on baseline memory (β=−6.04E-04, P=0.02), whereby it predicted baseline memory among Aβ+ individuals (β=0.44, P=0.01) but not among Aβ− individuals (β=0.06, P=0.46). Excluding *APOE* from PRS resulted in mainly LOAD PRS associations attenuating, but notably the resilience PRS interaction with CSF Aβ and selective prediction among Aβ+ individuals was consistent. Although the resilience PRS is currently somewhat limited in scope from the phenotype’s cross-sectional nature, our results suggest that the resilience PRS may be a promising tool in assisting in preclinical disease risk prediction among dementia-free and Aβ+ individuals, though replication and fine-tuning are needed.

## 1. Introduction

Late-onset Alzheimer’s disease (LOAD) is a highly polygenic disorder, characterized by a neuropathological cascade resulting in neurodegeneration and cognitive impairment.^1^ Notably, LOAD is characterized by a long prodromal phase in which pathology begins to accumulate prior to the onset of clinical disease. The prodromal stage thus represents decades of pathological changes before cognitive deficits are detected (e.g., dementia), making early clinical dementia diagnosis quite challenging,^1^ yet imperative. Additionally, LOAD is a highly heritable trait, with twin studies estimating LOAD heritability to be 60–80%,^2,3^ though the source for much of the genetic variation driving LOAD heritability has yet to be elucidated.^2,3^ Genome-wide association studies (GWAS) have been integral in beginning to uncover narrow-sense heritability, defined as the additive genetic component of heritability. As of 2022, LOAD GWAS have identified and replicated 33 risk and protective loci.^2,4^ However, the effect sizes of known LOAD GWAS loci are small to moderate,^5^ accounting for ~8% of total LOAD heritability, with ~6% out of this ~8% coming from the *APOE* ε2 and ε4 risk and protective alleles.^5^ Furthermore, studies have estimated the portion of LOAD narrow-sense heritability driven by common variants in the population, including and in addition to *APOE*. For example, Ridge and colleagues calculated that ~30% of LOAD phenotypic variance can be explained by a summation of effects of common GWAS variants,^5^ suggesting a substantial heritable component remains unexplained or missing.

In recent years, LOAD polygenic risk scores (PRS) have leveraged the effects of multiple genetic loci to predict LOAD risk, but these PRS have not had expected clinical utility. One reason is that LOAD PRS are often built from case/control GWAS, which may represent later-stage disease processes, resulting in a loss of sensitivity when applied to preclinical disease.^6^ Thus, LOAD PRS may be most beneficial in identifying symptomatic MCI or LOAD cases.^6^ At the same time, some studies have found that LOAD PRS can be built in a sensitive manner to predict MCI or LOAD risk in younger, dementia-free individuals.^7,8^ Yet, it also remains unclear if LOAD PRS hold more predictive power than simply predicting genetic risk from *APOE* genotype alone. Many studies have found that LOAD PRS hold predictive power for LOAD risk above and beyond *APOE* genotype,^9,10^ while other studies have found that *APOE* genotype is still the best predictor.^6,11^

While neuropathology is a hallmark of LOAD and other related disorders, it is notable that a subset of individuals can maintain normal cognition in the face of neuropathology. In fact, ~30% of elderly adults who meet NIA-AA Reagan neuropathological criteria for AD at autopsy remain cognitively unimpaired throughout life.^12,13^ These elderly individuals are characterized as “resilient” in frameworks of cognitive reserve and resilience.^14,15^ Our group has defined a continuous measure of resilience, representing better-than-expected cognition given amyloid pathology burden, and leveraged this measure for genomic analysis.^16^ The purpose of our original resilience GWAS was to identify common genetic variants that relate to cognition in the face of amyloid. By design, the residual metric of resilience is not correlated with amyloid, but is strongly predictive of future memory performance among people who are Aβ+.^16,17^ Notably, we found resilience to be 20–25% heritable,^17^ and found it has a genetic architecture distinct from that of clinical AD.^17^

However, to our knowledge, very few studies have examined polygenic resilience scores for complex traits, but these few have laid a framework for polygenic resilience scores as a tool to study complex, heritable traits. In 2021, Hess and colleagues created a method of calculating a “polygenic resilience score” for schizophrenia. In brief, this method takes marginal SNP effects from a trait, builds a weighted summary score from these SNP associations, and then selects the controls and the cases with the highest scores.^18^ Hou and colleagues applied this method to look at LOAD in the context of resilience and observed that a higher polygenic resilience score was associated with lower LOAD risk penetrance among high-risk LOAD individuals.^19^ A caveat of Hou and colleague’s study is that their findings attenuated when only examining their score among high-risk *APOE* ε4 carriers, and Hou et al. reiterates that PRS contributions above and beyond *APOE* is mixed in the literature.^19^ Additionally, a limitation of this polygenic resilience score method is that it uses trait-based GWAS and binning to determine “resilient” individuals, limiting the scope of the analysis.

We felt we could extend the polygenic resilience score framework by 1) leveraging our continuous, quantitative resilience phenotype 2) clarifying if a resilience PRS could predict risk above and beyond *APOE* 3) examine the relationship of a resilience PRS with amyloid pathology, which has been scarcely analyzed in LOAD PRS studies. Thus, we generated a LOAD^4^ PRS and a cognitive resilience^17^ PRS. In a dementia-free cohort, we assessed the association of each PRS with baseline memory and with annual memory decline and tested to see if amyloid modified the association of each PRS with memory performance. We hypothesized that while the LOAD PRS would be useful in predicting annual memory decline due to neuropathological build up, the resilience PRS would be more predictive of baseline memory in the presence of amyloid pathology, by differentiating the heterogeneity in memory performance among Aβ+ individuals.

## 2. Methods

### 2.1. Participants

Participants were recruited as part of a case-control, longitudinal, observational design study, the Vanderbilt Memory and Aging Project (VMAP) which takes place at the Vanderbilt University Medical Center in Nashville, Tennessee.^20^ VMAP began in 2012 and recruited individuals who were 60+ years of age, English speakers, had auditory/visual capacity for testing, and had a study partner. Each participant was given a Clinical Dementia Rating (CDR) interview and NIA-AA criteria was leveraged to classify individuals into cognitively unimpaired or mild cognitive impairment (MCI).^20^ All protocols for the VMAP cohort were IRB-approved and informed consent for each participant was obtained prior to enrollment. Please see Table 1 for an overview of the VMAP cohort.

### 2.2. Cerebrospinal fluid amyloid

A subset of participants (N=155) consented to and successfully completed lumbar puncture. Cerebral spinal fluid (CSF) was collected, spun down, and supernatant was analyzed through enzyme-linked immunosorbent assays (ELISA). One assay conducted was the INNOTEST^®^ β-AMYLOID_(1–42)_, which includes autoantibodies for neo-epitopes of amino acids 1 and 42 of the Aβ1–42 amino acid peptides, ensuring specificity for Aβ1–42 peptides. Binarized amyloid status was determined for each participant based on CSF Aβ1–42 measurements. A published cut-point of CSF Aβ1–42 530ng/L was implemented, thus defining Aβ+ individuals with CSF Aβ1–42 values under 530ng/L.^21^ A more detailed protocol is described in a prior paper by our group.^20^

### 2.3. Neuropsychological composites

Participants completed a series of neuropsychological tests that covered domains including memory, and a memory composite score was defined in a prior paper by our group.^22^ Memory composites were calculated from item-level data, to reduce multiple testing burden. The composite score leveraged test item-level data from the California Verbal Learning Test, Second Edition, and the Biber Figure Learning Test. Composite scores were calculated with a bifactor latent variable model, and final memory composite scores were on a z-score scale.^22^

### 2.4. Genetic data quality control and imputation

Individuals consenting to genotyping (N=333) were genotyped from whole blood on the Illumina MEGA^EX^ genotyping array. Raw genetic data were processed as follows. First, variant-level filtering removed variants with >5% missingness, <1% minor allele frequency (MAF), and non-autosomal variants. Next, sample-level filtering removed individuals with >1% missingness, those who were related, those with mismatched self-reported and genetically determined sex, and heterozygosity outliers. Then genetic data were filtered to keep self-reported non-Hispanic white individuals, and genetic ancestry outliers (e.g., principal component analysis – PCA) were removed. Variants were also filtered for Hardy-Weinberg equilibrium (HWE) exact test P<1×10^−6^. Finally, genetic data were lifted over to hg38 and compared and aligned to the Trans-Omics for Precision Medicine (TOPMed) reference panel,^23–25^ dropping variants that failed lift-over or mismatched with the reference panel.

Cleaned genetic data were next phased (Eagle phasing) and imputed on the TOPMed imputation server.^23–25^ Raw, imputed data were filtered to remove variants with an imputed R^2^<0.8 or duplicated/multi-allelic variants. Additionally, original genotypes were merged back in with the imputed data. Another HWE exact test was performed filtering for P<1×10^−6^, and variants with MAF <1% were removed. Once again, genetic ancestry outliers determined by a PCA were subsequently filtered. The final, cleaned, imputed VMAP genetic data included 255 non-Hispanic white participants and 8,689,730 variants. Additionally, *APOE* genotypes were determined by the TaqMan genotyping assay for rs7412 and rs429358 performed on DNA extracted from whole blood.^20^

### 2.5. Statistical analyses

**Figure 1.**
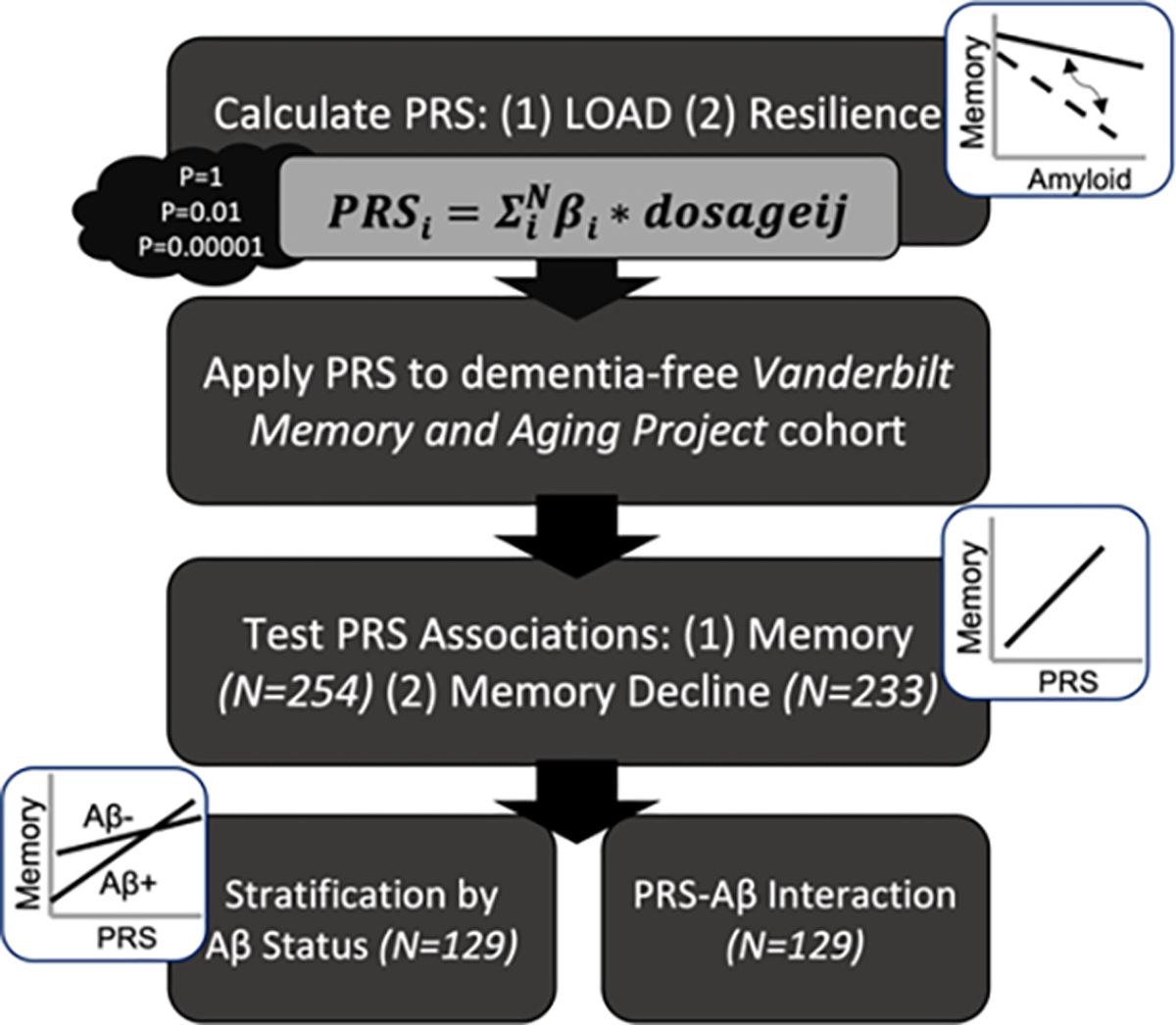
Flow-chart summary of analytical workflow.

See Figure 1 for an overview of our analytical plan.

#### 2.5.1. Polygenic risk score generation

Two PRS were calculated leveraging Kunkle et al. LOAD case/control genome-wide meta-analysis^4^ and our group’s recent genome-wide meta-analysis on resilience^17^. No participants in VMAP were included in either of the original GWAS. First, when applicable, GWAS were lifted to hg38. Next, GWAS variants were compared to the VMAP genetic data. Any ambiguous, palindromic variants were filtered out. Then overlapping variants between the GWAS and the VMAP genetic data were retained and then were compared for variants on opposite strands between the GWAS and the genetic data, and strand differences were resolved. Then, linkage disequilibrium (LD) clumping was performed with PLINK^26^ in the VMAP genetic data (r^2^=0.5, window=250kb), to choose the variant with the most significant phenotypic association within each genetically-linked genomic region. Each PRS was built with three different P-value thresholds: P=1, P=0.01, and P=0.00001, wherein variants were included in the PRS only if their phenotypic association was less than the given threshold. The LD-clumped genetic data were then leveraged to calculate each PRS with PLINK’s profile function^27^ which calculates scores as follows: Weights were retrieved from the variant associations with LOAD or with resilience from the respective GWAS. For each variant the given weight was multiplied by 0, 1, or 2, based on how many risk alleles an individual had. The summation of this process results in a summary score for an individual. Since *APOE* polymorphism is a robust risk factor for LOAD, PRS were calculated with and without the *APOE* region, defined by a 1Mb region up- and downstream of the *APOE* gene.

#### 2.5.2. Baseline and longitudinal linear models

We performed a series of linear models and linear mixed effects models in R (v. 4.2) for each PRS. Fixed effects in our models included baseline age, self-reported sex, and the given PRS. Linear mixed effects models included a PRS-by-interval term, where interval was determined by the difference between a participant’s age at each cognitive visit and their baseline age. Additionally, linear mixed effects models allowed slope and intercept to vary for each participant. In addition, we performed identical sets of models with the addition of a PRS-by-amyloid term in linear models and a PRS-by-amyloid-by-interval term for linear mixed effects models, with amyloid measured by the CSF Aβ1–42 assay outlined above. The outcome of our models were baseline memory or annual memory decline for linear models and linear mixed effect models, respectively. Each set of models above was performed again stratifying by amyloid status. Sensitivity analyses were performed for all models leveraging PRS generated without the *APOE* region.

## 3. Results

**Figure 2.**
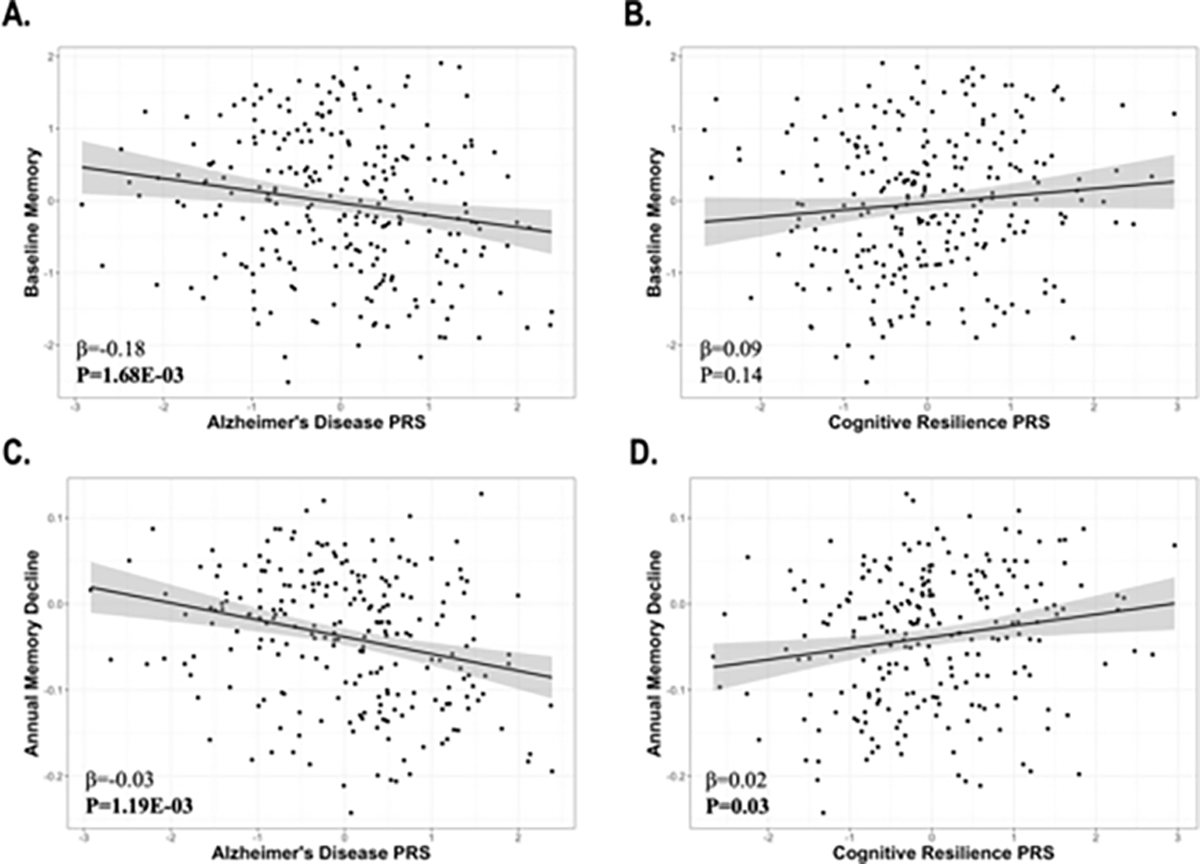
Main effect PRS associations (P=0.01 threshold; with *APOE*) with baseline memory (A, B) and annual memory decline (C, D).

We performed a series of linear models and linear mixed effects models investigating each PRS association with baseline memory or annual memory decline, respectively. All main effect associations are presented in Figure 2 and/or Table 2. The LOAD PRS had a significant main effect on baseline memory (Figure 2A; Table 2), but when *APOE* was excluded from the PRS, this result attenuated to nonsignificant (Table 2). Both the LOAD PRS (Figure 2C; Table 2) and the resilience PRS (Figure 2D; Table 2) had significant main effects on annual memory decline irrespective of *APOE* inclusion in PRS.

**Figure 3.**
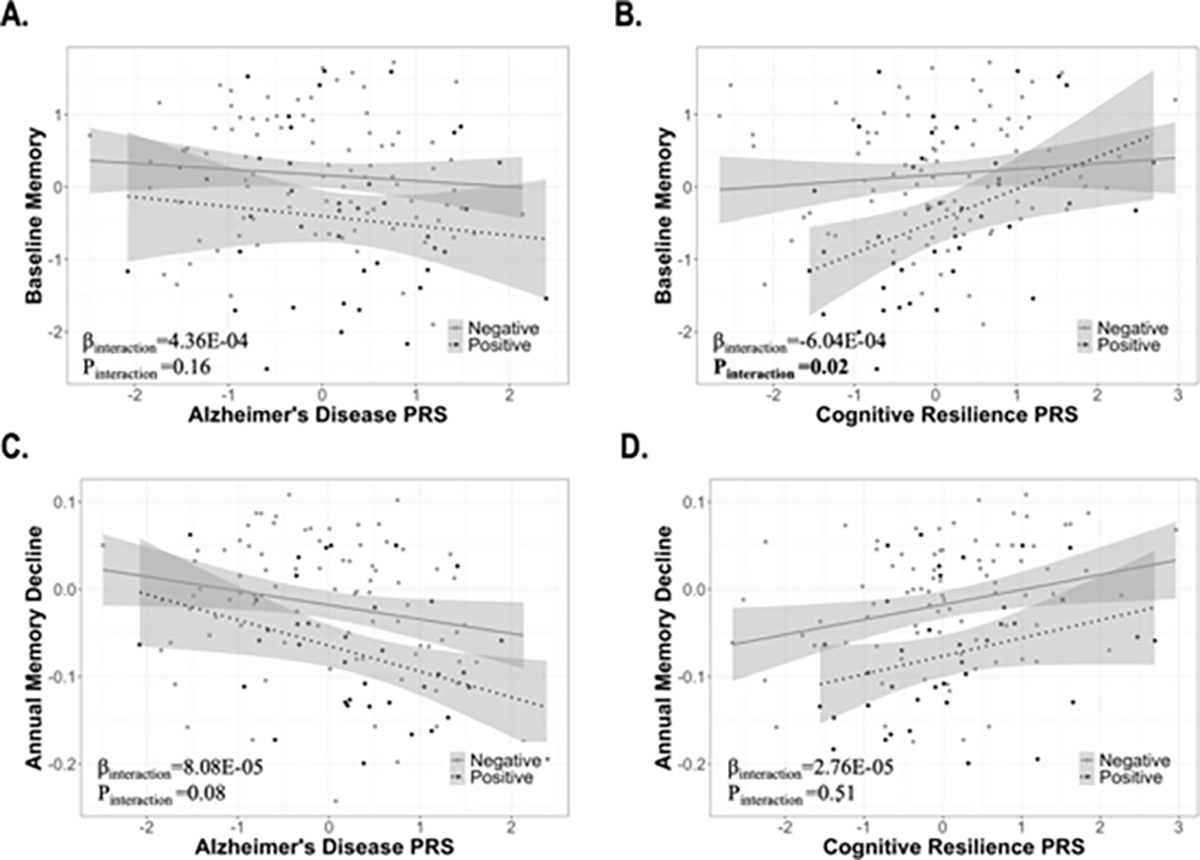
PRS associations (P=0.01 threshold; with *APOE*) with baseline memory (A, B) and annual memory decline (C, D) stratified by Aβ status.

Next, we performed a second series of models with a PRS-by-CSF-Aβ interaction term to determine if amyloid modified the association of each PRS with memory performance. Additionally, we performed amyloid status-stratified models to determine if Aβ− individuals or Aβ+ individuals (or neither) were driving any observed significant interactions. All CSF-Aβ interaction and amyloid-status stratified results are presented in Figure 3 and/or Table 2.

The LOAD PRS did not interact with CSF Aβ on either baseline memory (Figure 3A; Table 2) or annual memory decline (Figure 3C; Table 2), and this was consistent when *APOE* was excluded from PRS (Table 2). However, the LOAD PRS significantly predicted annual memory decline more strongly among Aβ+ individuals (Figure 3C; Table 2), albeit this result is difficult to interpret with the PRS-by-CSF-Aβ interaction term being nonsignificant. The resilience PRS significantly interacted with CSF Aβ on baseline memory (Figure 3B; Table 2), whereby it significantly predicted baseline memory among Aβ+ individuals (Figure 3B; Table 2) but not among Aβ− individuals (Figure 3B; Table 2). These results remained consistent when *APOE* was excluded.

In addition to the PRS with a P=0.01 threshold which are presented in the figures, we tested two other P-value thresholds: P=1 and P=0.00001 (Table 2). All results were consistent across all three thresholds unless denoted in the following paragraph. The LOAD PRS without *APOE* fell just under significance in the main effect association on annual memory decline at the P=1 and P=0.00001 thresholds. The resilience PRS did not have a main effect on annual memory decline at P=1 or P=0.00001 (with or without *APOE*). Additionally, the resilience PRS-by-CSF-Aβ interaction trended significant at P=1, but still significantly predicted baseline memory among Aβ+ individuals. Lastly, both the LOAD PRS and the resilience PRS varied by threshold – and by *APOE* inclusion for the LOAD PRS – in predicting annual memory decline among Aβ− individuals and/or among Aβ+ individuals.

## 4. Discussion

We built a LOAD PRS and a cognitive resilience PRS and evaluated each PRS in predicting memory outcomes among dementia-free elderly individuals. Both sets of PRS provided useful information and performed best in the spheres most closely related to the original phenotype in the GWAS. The LOAD PRS was predictive of annual memory decline in the whole sample and more strongly among Aβ+. In contrast, the resilience PRS was a particularly strong predictor of baseline memory in the presence of amyloid pathology, reflecting that the original phenotype was built to represent better-than-expected memory performance among those with high levels of AD biomarkers. Together, our findings suggest that the complementary information of a resilience PRS could improve preclinical prediction. It also highlights the need to expand sample sizes allowing for incorporation of longitudinal cognitive data into genetic studies of resilience to improve polygenic risk score applications in the future.

### 4.1. LOAD PRS is a strong predictor of annual cognitive decline in later stages of disease

Our main effect findings (Figure 2; Table 2) highlight that the LOAD PRS had a significant main effect on both baseline memory and annual memory decline. While the LOAD PRS did not interact with CSF Aβ on baseline memory or annual memory decline (Figure 3; Table 2), it more strongly predicted annual memory decline among Aβ+ individuals. LOAD PRS associations with cognitive decline have been replicated in other studies. For example, Kauppi and colleagues found that an AD PRS significantly predicted cognitive decline in a cohort of cognitive unimpaired individuals.^28^ Ge and colleagues determined that a LOAD PRS predicted cognitive decline among Aβ+ cognitively unimpaired and MCI individuals.^29^ Likewise, both Tan et al. and Desikan et al. observed that a polygenic hazard score was associated with cognitive decline.^30–32^ More specifically, Tan et al. found that those that had a high polygenic hazard score, indicative of high polygenic risk for LOAD, and who were Aβ+, showed steeper cognitive decline.^30–32^ Taken together, it may be that the LOAD PRS reflects a number of heterogeneous routes to cognitive impairment that includes AD neuropathology, but also includes some non-AD processes. All the studies mentioned as well as ours, found consistent associations with cognitive decline and stronger associations among Aβ+ individuals than among Aβ− individuals, though the difference in our non-demented cohort was negligible at best. It is notable that we did not observe a LOAD PRS-Aβ interaction. Perhaps the LOAD PRS models later stages of disease where Aβ accumulation has already occurred in many individuals and is but one contributor, while other pathways downstream and parallel to amyloidosis are primarily contributing to cognitive decline. This idea was posited by Carrasquillo and colleagues^6^ (and others) and appears to be supported by our findings.

### 4.2. Resilience PRS is a strong predictor of cognition in earlier stages of disease

Only the resilience PRS significantly interacted with Aβ on memory performance, whereby it predicted baseline memory among Aβ+ individuals but not among Aβ− individuals. (Figure 3; Table 2). Notably, previous studies are mixed regarding if LOAD polygenic risk associates with Aβ burden. Multiple studies have found associations between LOAD PRS and amyloid positivity, including Mormino and colleagues who also observed an association between their LOAD PRS and cognitive decline.^7,27,33^ Other studies have found no association between a LOAD PRS and amyloid positivity, or an association that attenuated when *APOE* was excluded.^11,29,34,35^ It is noteworthy that Ge and colleagues found no association between the LOAD PRS and baseline Aβ, but did find an association of the LOAD PRS with cognitive decline among Aβ+.^29^ Ebenau and colleagues comment on the mixed literature surrounding LOAD PRS association with Aβ positivity, pointing to heterogeneity in Aβ progression across diagnostic status as a potential reason for disagreement.^33^

Our original resilience phenotype was designed to predict better-than expected cognition in the presence of amyloid pathology.^16^ This matches what we are seeing with the resilience PRS, and the cross-sectional result we see with the PRS matches the cross-sectional nature of the phenotype.^16^ A recent study showed that a LOAD PRS enriched for amyloid-positivity-associated loci was associated with cognitive decline, whereas simply a LOAD PRS was not associated.^36^ This highlights that loci driving amyloidosis, which begins earlier in disease progression, may not be the same loci driving clinical dementia (downstream).^36^ To address this limitation, the resilience PRS may be a complementary tool in this case, as based on our novel results, it can selectively predict baseline memory among Aβ+ individuals (Figure 3; Table 2). Since much of the elderly population is living with neuropathology,^12^ determining those most at risk for future cognitive decline is imperative. Whereas the LOAD PRS may be working through amyloid pathology, performing similarly irrespective of amyloid pathology, the resilience PRS, in contrast, may be interacting with amyloid pathology, predicting genetic risk above and beyond amyloid pathology. It is noteworthy that all individuals in the VMAP cohort were dementia-free. Thus, our resilience PRS may be a tool that can best predict genetic risk for cognitive deficits among biomarker-positive individuals while they are still in the preclinical stage of disease. Our promising initial results indicate that we may have developed a novel PRS that 1) does not lose predictive power among those with Aβ pathology 2) performs its best among this high-risk Aβ+ group, separating them out from those in the elderly population who may or may not have Aβ in their brain, and 3) performs robustly irrespective of an individual’s future clinical diagnosis. Replicating our findings, incorporating longitudinal data into resilience models, and increasing sample size will be necessary to fine-tune this PRS.

### 4.3. PRS including more variants may have predictive power beyond the APOE locus

Over the last decade of PRS as a tool for LOAD risk prediction, there has been much debate regarding if a LOAD PRS has more predictive power than *APOE* genotype alone. Studies have been mixed, with many demonstrating that LOAD PRS associate with LOAD risk and LOAD-endophenotypes above and beyond *APOE*,^9,27,37^ while some studies show that LOAD PRS without *APOE* attenuate to nonsignificant in predicting LOAD risk or endophenotype levels.^11,29,34^ However, some of the studies that found PRS to contribute to risk prediction beyond that of *APOE* still underscore that *APOE* is contributing a large amount to polygenic risk.^9,37^ One study positing that a LOAD PRS has predictive power beyond *APOE* also stated that 43.8% of the 61.0% total predictive power of the LOAD PRS on conversion from MCI to LOAD was coming from *APOE* alone.^9^ Notably, our resilience PRS findings remained consistent when *APOE* was removed from PRS calculations, which makes sense as the resilience phenotype attempts to regress out effects of amyloidosis^17^ which are often driven by *APOE*.^34^ A resilience PRS like the one we built in this study may be promising in terms of its ability to predict LOAD-related cognitive outcomes above and beyond that of *APOE* but replicating our findings and larger sample sizes for future resilience GWAS are needed to fully elucidate this theory.

In addition, there is no gold standard for a singular P-value threshold to leverage for LOAD PRS calculations. Two recent studies examined LOAD PRS at a variety of different thresholds. Ge and colleagues observed fairly consistent results across thresholds spanning from P=0.01 to P=1×10^−7^.^38^ Another study observed that distinguishing between cognitively unimpaired and LOAD participants was best with a threshold of P=0.01, and in fact predictive power plateaued after P=0.01.^27^ In this study, we tested three thresholds: P=1, P=0.01, and P=0.00001. Our results were mostly consistent across the three thresholds, but the resilience PRS at P=0.01 seemed to best predict annual memory decline. Overall, our results combined with some previous studies suggest that perhaps allowing for inclusion of more loci that fall below the stringent genome-wide threshold captures a wider variety of processes contributing to complex trait risk.^18,19,27^

### 4.4. Strengths and weaknesses

Our study had multiple strengths. We leveraged a deeply-phenotyped cohort, the Vanderbilt Memory and Aging Project. This cohort has many important features including participants free of dementia, baseline biomarker status for participants, and longitudinal measurements of memory composite scores. However, our study did have some limitations. Our resilience PRS was not built with inclusion of measures of tau pathology or other known age-related neuropathologies. Sample size is a limiting factor for these measures of pathology, but as sample sizes increase in these cohorts, we plan to incorporate other pathology measures into our resilience models in addition to amyloid. Additionally, our sample size (in VMAP) was limited to those consenting to genotyping, neuropsychological testing, and lumbar puncture. Our study was limited to non-Hispanic white individuals, attenuating the generalizability of our findings to other populations. Currently, genetic data is becoming available for individuals across multiple ancestry groups, allowing groups including ours to expand diversity in GWAS studies, including cross-ancestry approaches. With more diverse GWAS, future studies will be able to build PRS in multiple ancestry groups, which will aid in our understanding of AD genetic risk in diverse populations. Lastly, some of the PRS associations reported in this study did not survive correction for multiple comparisons with the false discovery rate (FDR<0.05) procedure, likely due to power and sample size constraints of the original GWAS. The sample sizes of individuals with cognition, genotyping, and neuropathology data are ever increasing, which we are leveraging to increase our sample sizes for our resilience GWAS, and this will contribute to increased power in an analysis like this one in the future.

### 4.5. Conclusions

Although our study needs to be replicated, we find our initial novel findings to be promising that a cognitive resilience PRS may serve as a complementary clinical tool with a LOAD PRS in identifying those most at risk for future cognitive decline while individuals are still in the preclinical and prodromal stages of LOAD.

## Figures and Tables

**Table 1.** VMAP Cohort Demographics.

Cohort Characteristics	

Number of participants	334
Number of participants with genetic data	76.05% (254)
Total number of visits	3.83 +/− 0.76
Longitudinal follow-up (years)	2.27 +/− 1.97

Demographics and Health Characteristics	

Age at baseline (years)	72.74 +/− 6.89
Sex (% female)	27.54% (92)
Education (years)	16.13 +/− 2.56
*APOE* ε4 (% positive)	26.35% (88)
Amyloid status (% positive)	49.70% (166)
Diagnosis at baseline (% MCI)	29.34% (98)

**Table 2.** PRS Associations with Baseline Memory and Annual Memory Decline.

**Baseline Memorv**									

***PRS***		***Main Effect***		Aβ*PRS		***Aβ−***		***Aβ+***	

PRS	Threshold	β	P	β	P	β	P	β	P

LOAD	P=1	−0.13	***0.03***	2.73E-04	0.41	−0.11	0.21	−0.10	0.59
LOAD	P=0.01	−0.18	***1.68E-03***	4.36E-04	0.16	−0.08	0.37	−0.13	0.47
LOAD	P=0.00001	−0.25	***4.00E-05***	5.70E-04	0.13	0.02	0.87	−0.11	0.57
Resilience	P=1	0.09	0.12	−4.97E-04	0.08	0.10	0.21	0.57	***1.33E-03*** *
Resilience	P=0.01	0.09	0.14	−6.04E-04	***0.02*** *	0.06	0.46	0.44	***0.01*** *
Resilience	P=0.00001	0.01	0.88	−7.83E-04	***0.02*** *	−0.09	0.30	0.52	***0.02*** *

**Annual Memory Decline**									

***PRS***		***Main Effect***		Aβ*PRS		***Aβ−***		***Aβ+***	

PRS	Threshold	β	P	β	P	β	P	β	P

LOAD	P=1	−0.02	***0.02***	2.59E-05	0.63	−0.03	***0.04*** *	−0.05	***0.01*** *
LOAD	P=0.01	−0.03	***1.19E-03*** *	8.08E-05	0.08	−0.03	*0.09* ^#^	−0.05	***0.01*** *
LOAD	P=0.00001	−0.03	***8.66E-04***	7.16E-05	0.21	−0.01	*0.54* ^#^	−0.02	0.23
Resilience	P=1	4.74E-03	0.60	3.66E-05	0.42	9.88E-04	0.94	0.04	***4.69E-02*** *
Resilience	P=0.01	0.02	***0.03*** *	2.76E-05	0.51	0.03	***0.02*** *	0.02	0.36
Resilience	P=0.00001	0.01	0.39	−6.30E-05	0.25	−2.41E-03	0.87	0.08	***6.60E-04*** *

Note: P-values with * remain significant without APOE; # significant without APOE only
